# Advanced robotic liver surgery

**DOI:** 10.1007/s00464-026-13093-w

**Published:** 2026-07-06

**Authors:** Jacopo Mascherini, Cristiano Guidetti, Hasan Al Harakeh, Roberta Odorizzi, Paolo Magistri, Gian Piero Guerrini, Iswanto Sucandy, Fabrizio Di Benedetto

**Affiliations:** 1 Digestive Health Institute, AdventHealth Tampa, Tampa, FL USA; 2 Hepato-Pancreato-Biliary Surgery and Liver Transplantation Unit, University Hospital of Modena “Policlinico”, University of Modena and Reggio Emilia, 41124 Modena, Italy; 3 Department of Abdominal Organ Transplantation, University of Arizona, Tucson, AZ USA

**Keywords:** Robotic liver surgery, Minimally invasive hepatectomy, Complexity, Hepatobiliary surgery, Difficulty scoring systems, Robotic-assisted surgery

## Abstract

**Background:**

Robotic surgery represents the most advanced evolution of minimally invasive liver surgery, progressively extending its application from minor resections to highly complex hepatobiliary procedures. Beyond the traditional definition of major hepatectomy, surgical complexity is increasingly determined by anatomical location, proximity to major vascular and biliary structures, patient-related factors, and integration within multimodal treatment strategies.

**Methods:**

A narrative and critical review of the literature was conducted using PubMed/MEDLINE, focusing on robotic liver resections performed in technically complex settings. Case series, original studies, meta-analyses, consensus statements, and guidelines from high-volume centers with expertise in minimally invasive liver surgery were preferentially included.

**Results:**

Current evidence supports the technical feasibility and safety of robotic liver surgery across multiple domains of complexity, including resections of posterosuperior segments, parenchyma-sparing procedures for centrally located tumors, vascular and biliary resections with reconstruction, and resections following liver volume augmentation strategies. Selected experiences also demonstrate feasibility in extreme scenarios such as robotic living donor hepatectomy and liver transplantation. Compared with open surgery, robotic approaches are associated with reduced surgical trauma and shorter hospital stay. Potential advantages over laparoscopy are mainly technical and procedure specific. Long-term oncological superiority has not been conclusively demonstrated in the current literature.

**Conclusions:**

Robotic liver surgery has matured into a versatile platform capable of supporting highly complex hepatobiliary procedures in carefully selected patients. Its value lies in enhancing technical precision and facilitating minimally invasive procedures within established oncological and transplant indications. Widespread adoption should remain limited to specialized, high-volume centers with structured training and multidisciplinary expertise.

Robotic surgery stands out as the most advanced expression of minimally invasive surgical approaches. The platform’s well-known unique characteristics inspired the scientific community to explore the boundaries of current surgical practice and redefine what can be safely and effectively performed through minimally invasive techniques. Liver surgery makes no exception to this paradigm shift. Since the first series of robotic liver resections (RLR) was reported by Giulianotti et al. in 2003 [1], many centers around the world have integrated robotic technology into hepatobiliary surgical practice. As surgical techniques, knowledge, and technology have evolved, procedures previously considered unsuitable for minimally invasive approaches have increasingly been accomplished and reported with the robotic platform (Table 1).

**Table 1:** Milestones in robotic liver surgery

Authors	Milestone	Year
Giulianotti et al. [1]	1st robotic hepatic resection	2003
Giulianotti et al. [2]	1st robotic right extended hepatectomy with biliary reconstruction (perihilar cholangiocarcinoma)	2010
Giulianotti et al. [3]	1st robotic right lobe living donor hepatectomy	2012
Vicente et al. [4]	1st robotic ALPPS	2016
Khan et al. [5]	1st fully robotic whole graft LT	2024
Broering et al. [6]	1st fully robotic LDLT	2024

*ALPPS* associating liver partition and portal vein ligation for staged hepatectomy, *LT* liver transplantation, *LDLT* living donor liver transplantation

Traditionally, the complexity of liver surgery has been defined according to the extent of parenchymal resection, with major hepatectomy described as the removal of three or more contiguous liver segments [7]. However, this definition alone does not adequately reflect the operative difficulty encountered in modern surgery. Technical complexity is often driven by additional factors, including lesion location within posterosuperior segments, proximity to major vascular and biliary structures, need for parenchyma-sparing strategies, underlying liver disease, and integration within multimodal or staged pathways.

Within this evolving framework, robotic technology offers potential advantages that extend beyond simple magnification or minimally invasive access. Enhanced dexterity, tremor filtration, stable three-dimensional visualization, and improved ergonomics may facilitate procedures characterized by fine dissection, deep parenchymal transection, and reconstructive phases. At the same time, the expansion of robotic indications raises critical questions regarding patient selection, training requirements, evidence quality, cost-effectiveness, and the limits of safe implementation.

The term “advanced robotic liver surgery” should therefore not be interpreted merely as the robotic replication of major hepatectomy, but rather as the application of robotic technology to high-complexity hepatobiliary scenarios in which technical difficulty becomes the primary determinant of operative risk.

The purpose of this narrative review is to summarize and discuss current evidence on advanced liver procedures performed using the robotic approach. By framing advanced robotic liver surgery within a multidimensional model of complexity, we aim to provide a structured overview of its current applications, limitations, and future directions.

## Methods

This article was designed as a narrative and critical review of the currently available literature addressing the role of robotic surgery in complex hepatobiliary procedures.

A structured literature search was conducted using PubMed/MEDLINE as the primary database. The search included articles published between January 2000 and January 2026. The search strategy included the following terms and their alternatives, combined using Boolean operators: robotic liver resection, robotic hepatectomy, posterosuperior segments, parenchyma-sparing liver resection, vascular reconstruction, difficulty score, biliary reconstruction, ALPPS, two-stage hepatectomy, liver venous deprivation, living donor hepatectomy, and robotic liver transplantation (LT).

Original studies, case series, comparative studies, systematic reviews, meta-analyses, consensus statements, and international guidelines were considered eligible if they reported technical aspects, perioperative outcomes, or oncologic data related to robotic liver surgery in complex settings. Publications not available in English and conference abstracts without full-text articles were excluded.

Given the narrative design of the review, study selection was guided by relevance to the predefined domains of surgical complexity and by the level of technical detail provided. No formal quantitative synthesis was performed. When available, priority was given to high-volume centers and studies reporting reproducible technical strategies.

## Definition of complex liver resection

Traditionally, *major* liver resection is defined as the removal of three or more contiguous liver segments according to Couinaud’s classification [7]. However, this definition alone fails to fully capture the degree of technical complexity of liver surgery. As a result, the concept of *technically major* resection has implicitly emerged to describe procedures characterized by high operative difficulty despite limited anatomical extent.

In the minimally invasive setting, this concept has gained even greater relevance [8]. Indeed, resections involving a limited volume of liver parenchyma may prove to be technically complex due to unfavorable lesion location, proximity to major vascular/biliary structures, or the need for reconstructive maneuvers.

To address these challenges, difficulty scoring systems (DSSs) have been developed to better stratify operative complexity. Despite some methodological differences (as summarized in Table 2), the most widely validated DSS [9–13] consistently identify the type of resection, lesion size, and lesion location as key factors determining liver resection complexity. While initially designed for laparoscopic approaches, these DSSs have also been shown to be applicable to the robotic setting [14]. In a recent international survey specifically addressing complex robotic liver surgery, procedures corresponding to advanced and expert grades of the Iwate classification (scores 7–12) were proposed as a pragmatic definition of complexity in the robotic setting [15].

**Table 2:** Summary of difficulty scoring systems for minimally invasive liver resection

Score (year)	Approach	Extent of resection	Lesion location	Lesion size	Liver function	Other variables	Externally validated
Ban (2014) [9]	LLR	Y	Y	Y	N	Proximity to major vessels	Y
Iwate (2016) [10]	LLR	Y	Y	Y	Y	Proximity to major vessels Hybrid procedure	Y
Hasegawa (2017) [11]	LLR	Y	Y	N	N	Obesity Platelet count	Y
IMM (2018) [12]	LLR	Y	N	N	N	None	Y
Southampton (2018) [13]	LLR	Y	N	Y	N	Previous open liver surgery Lesion type Neoadjuvant chemotherapy	Y
Tampa (2024) [16]	RLR	Y	Y	Y	N	Neoadjuvant chemotherapy Lesion type Portal lymphadenectomy Bile duct resection/reconstruction	Y

*LLR* laparoscopic liver resection, *IMM* Institute Mutualiste Montsouris, *RLR* robotic liver resection

While such a definition provides a useful standardized framework, it remains primarily anatomy-based and does not fully account for reconstructive complexity, parenchymal vulnerability, or integration within staged and multimodal treatment strategies. Furthermore, most DSSs were originally developed for laparoscopic surgery and may incompletely capture the specific technical features and potential advantages of the robotic platform [16]. For this reason, procedure-specific scoring systems for RLR, such as the Tampa Difficulty Score, have been developed and externally validated [17, 18].

In the context of advanced robotic liver surgery, complexity should therefore be regarded as a multidimensional construct encompassing:

1. Anatomical difficulty related to tumor location and vascular proximity;
2. Technical demands including vascular or biliary resection and reconstruction;
3. Parenchymal quality and patient-related risk factors;
4. Integration of liver resection within staged hepatectomy, regenerative strategies, or transplant-related procedures.

This multidimensional perspective provides a conceptual framework for interpreting complexity in robotic liver surgery.

## Patient- and context-related determinants of operative complexity

Beyond procedural factors, operative complexity of RLR is also influenced by patient- and context-related features.

Cirrhosis and portal hypertension reportedly increase postoperative morbidity and mortality [19, 20]. In cirrhotic patients, evidence specifically addressing RLR showed clear benefits compared to open resections, even in advanced stage hepatocellular carcinomas [21, 22].

Additional factors potentially increasing operative complexity include obesity, prior chemotherapy, and previous abdominal surgery. In obese patients—especially in those with a narrow ribcage—exposure and operative field may be substantially reduced. Obesity has been shown to be associated with worse postoperative outcomes after open and laparoscopic liver resection [23]. Furthermore, Hasegawa et al. reported a significant increase in operative time in patients with BMI > 30 kg/m^2^ undergoing laparoscopic liver resection; hence, obesity is included among the items of their DSS [11]. Also, the presence of fatty liver disease spectrum may impair parenchymal transection, increasing intraoperative blood loss [24]. Nevertheless, studies addressing the subpopulation of high BMI patients undergoing RLR report comparable outcomes to normal BMI patients, suggesting the safety of the robotic approach [25] in terms of complications and adequacy of resection. A recent multicenter retrospective analysis conducted on patients undergoing RLR found no significant differences in perioperative outcomes among obese and non-obese patients [26].

In a similar fashion, neoadjuvant chemotherapy regimens in the context of colorectal liver metastases may induce parenchymal injury, potentially reducing parenchymal quality and making parenchymal transection more demanding [27]. Moreover, this may lead to the requirement for a larger future liver remnant (FLR) to avoid post-hepatectomy liver failure (PHLF), leading to more articulated procedures or the necessity for FLR growth induction techniques.

Previous abdominal surgery can lead to adhesion formation, potentially requiring extensive adhesiolysis. However, although often regarded as a limiting factor for minimally invasive liver surgery, a systematic review on reasons for RLR conversion did not identify adhesiolysis as a reason for conversion [28]. Moreover, RLR performed in patients with previous abdominal surgery showed comparable outcomes to patients with no previous surgery [29]. In the context of repeat hepatectomy, RLR showed a significantly reduced conversion rate compared to traditional laparoscopy, and no conversion was due to extensive adhesiolysis in the robotic cohort [30].

Collectively, although mostly retrospective in nature, this evidence suggests that several patient- and context-related factors should not be regarded as absolute contraindications to RLR. In selected cases, the robotic platform may even help mitigate some of the technical challenges associated with these conditions.

## Technical domains of operative complexity

Despite this background, specific procedural scenarios can be delineated in which technical complexity emerges as the principal limiting factor in RLR.

### Posterosuperior liver resections

Resections involving posterosuperior segments (i.e., segments 4a, 7, 8, and the paracaval portion of segment 1) are widely regarded as among the most technically demanding procedures in liver surgery, especially in MILS. This is mainly due to close proximity to the inferior vena cava and major hepatic veins, and to their subdiaphragmatic position, all of which limit exposure and restrict the operative workspace. In this context, the increased freedom of movement and articulation of the robotic instruments may mitigate some of the intrinsic limitations of traditional laparoscopy [31].

Several studies have explored the feasibility and safety of RLR of lesions involving these segments. Nota et al. retrospectively compared robotic and open liver resections of the posterosuperior segments, reporting significantly shorter postoperative length of stay in the RLR group (median [interquartile range] of 4 [3–7] days versus 8 [6–10] days, *p* < 0.001) with similar complication rates [32]. More recently, comparative meta-analyses evaluating RLR and LLR for posterosuperior segments have suggested potential advantages of RLR in terms of blood loss, intraoperative transfusion rate, conversion rate, and operative time [33, 34]. Another meta-analysis comparing RLR and LLR reported lower intraoperative blood loss (mean difference − 107 mL [95% CI − 205 to − 9]) and shorter hospital stay (mean difference − 0.73 days [95% CI − 1.21 to − 0.25]) for RLR specifically in posterosuperior resections [35].

While these findings should be interpreted with caution, they support the notion that robotic assistance may expand the applicability of MILS to anatomically challenging liver segments in appropriately selected patients and in centers with established robotic expertise.

**Fig. 1:**
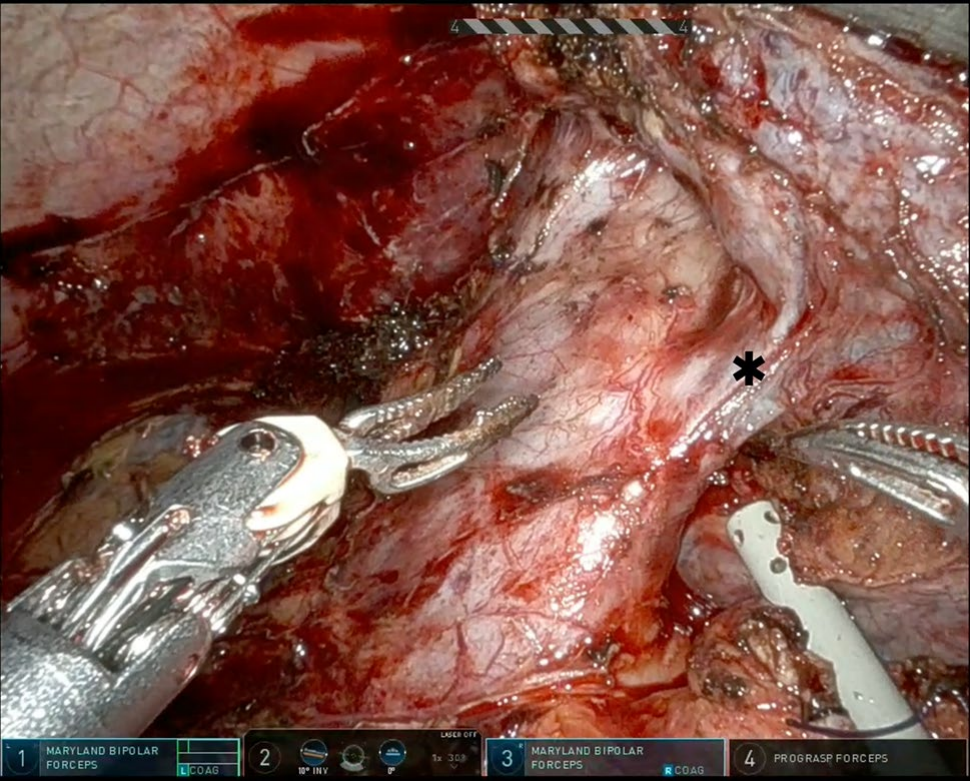
Inferior vena cava exposure. The liver is retracted cephalad and to the left by the fourth robotic arm, providing stable exposure of the caval plane. Maryland forceps on the third robotic arm are used to carefully dissect free a large right inferior hepatic vein (✱), which is subsequently divided

In our experience, one of the main advantages of RLR in this setting is the retraction provided by the fourth robotic arm, which enables stable lifting of the liver and facilitates the exposure of the caval plane during deep parenchymal transection and liver mobilization (Fig. 1). To optimize this, the fourth arm port should be placed slightly cranial to the main robotic trocar line, closer to the left inferior costal margin, thereby allowing a wider range of motion toward the diaphragm.

In addition, the TilePro® feature allowing for a precise intraoperative ultrasound within the robotic console is particularly valuable in this setting, as it helps to define the relationship between the lesion, the major hepatic veins, and the caval plane, thereby guiding a safer transection strategy despite limited exposure.

**Expert tips.** For posterosuperior resections, careful preoperative assessment of the relationship between the lesion, hepatic veins, and inferior vena cava is essential to plan exposure and the direction of transection. Intraoperatively, stable retraction with the fourth robotic arm, repeated intraoperative ultrasound assessment, and early readiness for inflow control are key safeguards when working in the subdiaphragmatic space or close to major hepatic veins.

### Central liver tumors and parenchyma-sparing resections

Malignant lesions located centrally within the liver parenchyma and in close proximity to major vascular and biliary structures have traditionally been managed with major or extended hepatectomies. However, the presence of underlying liver disease or insufficient FLR may limit resectability because of increased risk of PHLF. In this context, parenchyma-sparing resections such as central hepatectomy have been proposed as an alternative to extended chances of cure [36]. By preserving functional liver tissue, these technically challenging procedures have been associated with fewer complications compared to extended liver resections [37]. The feasibility of robotic central hepatectomy has been confirmed by reports of several case series [38–40].

Beyond central hepatectomy, more advanced parenchyma-sparing strategies have been developed with the aim of maximizing functional liver preservation while maintaining oncological radicality. These approaches include non-anatomical and ultrasound-guided tailored resections, often performed in close proximity to major vascular structures [41]. In advanced biliary malignancies, particularly when hilar or periarterial dissection is required, oncological radicality also depends on meticulous clearance of the periarterial lymphatic and neural tissue along the hepatic artery and its branches. The robotic platform may facilitate this step through stable magnified visualization and precise wristed dissection, provided that vascular control, exposure, and oncological planes are safely maintained. This parenchyma-sparing philosophy has been largely developed and refined in open liver surgery, where intraoperative ultrasound plays a pivotal role in guiding resection planes and preserving critical inflow and outflow [42]. Although the minimally invasive application of these resection strategies remains limited, selected principles can be transferred to minimally invasive liver surgery to support parenchyma preservation without compromising oncological radicality.

**Fig. 2:**
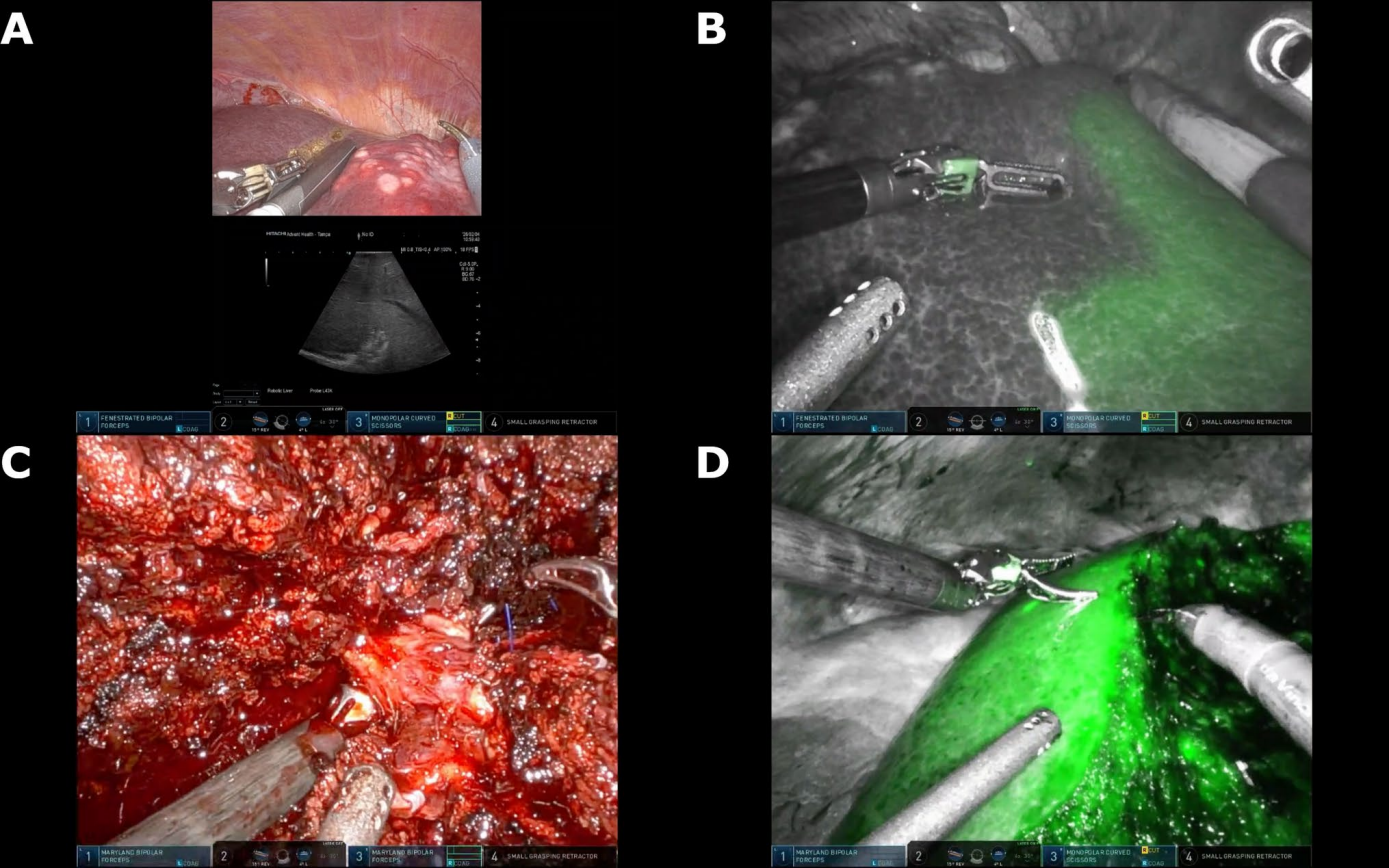
Right anterior sectionectomy (bisegmentectomy segments V and VIII) for a centrally located liver tumor. **A** Tumor margins and anatomical relationships with the surrounding vasculobiliary structures are assessed using intraoperative ultrasound. **B** The transection plane is refined using the indocyanine green negative staining technique. **C** During parenchymal transection, the anterior section Glissonean pedicle is identified and dissected free in preparation for division. **D** After completion of the resection, indocyanine green fluorescence is used to confirm adequate perfusion of the right posterior section (Color figure online)

In this context, intraoperative ultrasound remains the cornerstone of these approaches. The robotic platform may facilitate its use through the integrated display within the surgical console, allowing the operating surgeon to maintain continuous correlation between ultrasound findings and operative maneuvers. In addition, indocyanine green fluorescence imaging may provide a useful adjunct, particularly for anatomical orientation, segmental demarcation, and lesion detection (Fig. 2) [43].

More broadly, the robotic platform offers a favorable environment for the integration of adjunctive imaging technologies. Emerging tools such as image-guided navigation, artificial intelligence, and augmented reality may further enhance spatial understanding during complex parenchyma-sparing resections, and their future refinement may expand the role of robotics as an interface between surgical execution and real-time image guidance [44].

**Expert tips.** In central and parenchyma-sparing resections, the transection plane should be planned according to the intrahepatic vascular anatomy rather than the surface projection of the tumor. Repeated intraoperative ultrasound assessment, combined when appropriate with indocyanine green fluorescence, helps preserve functional inflow, outflow, and biliary drainage while maintaining oncologic adequacy.

### Vascular and biliary reconstruction

Some cases impose vascular or biliary resection and reconstruction. Achieving adequate oncological clearance in centrally located or advanced hepatobiliary malignancies may require en bloc resection of portal vein branches, hepatic veins, or biliary structures, followed by reconstruction to restore physiological inflow, outflow, or biliary drainage. Owing to the extreme grade of difficulty, these procedures have traditionally been regarded as a major limitation to MILS.

The feasibility of robotic surgery in this setting was initially demonstrated by Giulianotti et al., who described the first case of robotic extended right hepatectomy with biliary reconstruction for perihilar cholangiocarcinoma [45]. Subsequent case series from high-volume and highly experienced centers have progressively strengthened these initial observations, confirming the technical reproducibility and safety of robotic biliary reconstruction [46–50]. In a retrospective single-center analysis, Liu et al. reporting significantly faster hepato-jejunal anastomosis in RLR compared to LLR (mean ± standard deviation of 38.4 ± 13.6 min versus 59.1 ± 25.5 min, *p* = 0.024), with a numeric trend toward lower complication rates (bile leak rate 40% in LLR versus 10% in RLR, *p* = 0.204), suggesting that the robotic platform may enhance surgical performance in these technically demanding biliary anastomoses [51].

Beyond biliary reconstruction, selected reports have also described concomitant portal vein resection and reconstruction during robotic liver resections, further expanding the technical boundaries of the approach [52, 53]. A recently published article describes in detail the technique of tangential resection and reconstruction of the middle hepatic vein during central hepatectomy [54]. Furthermore, Wang et al. reported the first case of robotic hepatectomy with concomitant middle hepatic vein resection and reconstruction using an interposed synthetic graft [55]. These experiences highlight the potential role of robotics even in complex venous reconstructions.

**Fig. 3:**
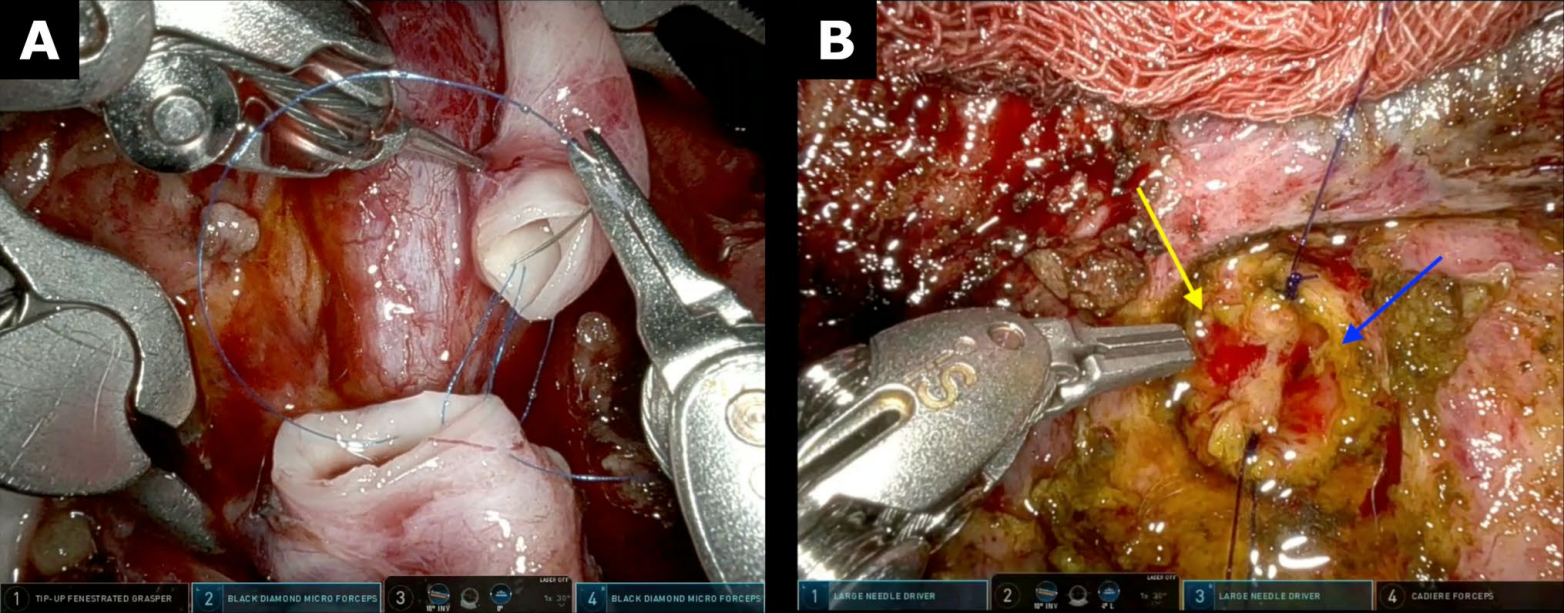
Representative examples of advanced robotic intracorporeal suturing. **A** End-to-end arterial anastomosis during robotic liver transplantation. The anastomosis is performed in a continuous fashion with the parachute technique, using a 7-0 Prolene suture and Black Diamond micro-forceps. **B** Bile duct plasty between right (yellow arrow) and left (blue arrow) bile ducts after bile duct resection, in preparation for a hepatojejunostomy. The plasty is performed in a continuous fashion using a 6-0 PDS suture (Color figure online)

At present, evidence supporting robotic vascular resection and reconstruction remains limited and largely confined to highly selected cases. Nevertheless, as feasibility has been established, ongoing refinements in surgical technique and perioperative management, together with advances in multimodal oncological treatment, may support a cautious expansion of current indications in highly selected settings. This perspective is also consistent with recent jury-based recommendations in robotic hepato-pancreato-biliary surgery, which identified advanced intracorporeal suturing as one of the scenarios in which the robotic platform may offer the most relevant technical advantages, particularly for vascular and biliary reconstruction (Fig. 3) [56]. In this context, the progressive development of robotic transplantation may further contribute to the maturation of these reconstructive skills in highly specialized hepatobiliary programs.

**Expert tips.** Vascular reconstructions were initially performed with fine expanded polytetrafluoroethylene (GORE-TEX; W. L. Gore & Associates, Newark, DE, USA) sutures for their increased resistance to traction. Later, non-absorbable monofilament polypropylene (Prolene; Ethicon, Inc., Somerville, NJ, USA) sutures were adopted due to their reduced traumatic effect on vessel walls. Biliary reconstructions were performed with absorbable monofilament sutures, either polydioxanone (PDS; Ethicon, Inc., Somerville, NJ, USA) or polyglyconate (Maxon; Covidien/Medtronic, Minneapolis, MN, USA).

### Resection after liver volume augmentation

Liver volume augmentation strategies can be employed to prevent PHLF when the predicted FLR is insufficient.

Portal vein embolization (PVE) is the suggested primary technique in most settings. In case of insufficient FLR hypertrophy after PVE, the use of double vein embolization/liver venous deprivation (DVE/LVD) is increasingly adopted as the first rescue procedure [57]. Randomized clinical trials comparing these two strategies are currently ongoing; therefore, the optimal choice remains under debate [58, 59]. Two-stage hepatectomy (TSH) is widely used in bilobar colorectal liver metastases. Typically, the first stage consists of the clearance of the FLR combined with embolization or ligation of the contralateral portal branch to induce hypertrophy before completion hepatectomy [57]. In patients with very small FLR, when radiological augmentation fails to achieve adequate FLR hypertrophy, or if portal vein thrombosis is present, associating liver partitioning and portal vein ligation for staged hepatectomy (ALPPS) can provide a reasonable rescue strategy [57].

Although the role of ALPPS has been reconsidered with the advent of alternative FLR augmentation strategies, the technical feasibility of the procedure using a robotic approach has been demonstrated across multiple clinical settings [60–65]. Current evidence supports a more selective use of surgical first-stage procedures and ALPPS, particularly in light of the increasing adoption of radiological FLR augmentation strategies. When required, a minimally invasive approach to the first stage of TSH and ALPPS is generally advocated, since it has been shown to limit adhesions at the second stage [66]. Furthermore, minimally invasive approaches have been employed in an attempt to reduce the high rates of morbidity and mortality associated with open ALPPS [67–69].

From a technical standpoint, parenchymal transection remains a key issue in robotic staged procedures. This is especially relevant in cases with pathologic liver parenchyma, such as fatty liver disease, chemotherapy injury, cirrhosis, or congestion after FLR augmentation strategies. In the absence of a dedicated robotic ultrasonic dissector, several expert centers have adopted hybrid strategies incorporating laparoscopic CUSA alongside robotic energy devices such as Harmonic Ace®, SynchroSeal®, and VesselSealer®. However, the use of CUSA should not be considered mandatory in robotic liver surgery. Increasing evidence and technical experience support the feasibility of fully robotic parenchymal transection techniques—such as clamp-crush or double-bipolar approaches—which can effectively replace the need for ultrasonic aspiration across a wide range of resection settings. Moreover, hybrid approaches inherently depend on the presence of a highly skilled table-side assistant, often with specific hepatobiliary expertise, potentially limiting their reproducibility. In contrast, the development and standardization of fully robotic techniques may enhance procedural autonomy and broader applicability beyond high-volume expert centers [15]. A large propensity score-matched multicenter analysis compared parenchymal transection techniques, reporting similar perioperative outcomes between a full robotic double-bipolar approach and the hybrid robotic-laparoscopic technique [70].

Initial experiences of RLR after PVE or DVE/LVD have been described [71, 72]. In this context, the combination of lower-morbidity radiological FLR augmentation strategies with the reduced invasiveness of RLR appears conceptually attractive in selected patients. However, its wider reproducibility will depend on further technical standardization, particularly with regard to robotic parenchymal transection.

**Expert tips.** In staged resections involving a surgical first stage, the initial operation should already be planned with the completion procedure in mind. Limiting unnecessary hilar dissection, preserving clean planes for subsequent mobilization, and choosing trocar placement that remains effective after FLR hypertrophy may facilitate second-stage hepatectomy and help preserve the advantages of the minimally invasive approach.

### Pushing the boundaries: robotic living donor hepatectomy and liver transplantation

Living donor hepatectomy (LDH) and LT combine demanding parenchymal transection, meticulous vascular and biliary dissection, and complex reconstructive phases within a time-sensitive and physiologically critical setting. Historically, these procedures have been considered beyond the scope of minimally invasive approaches because of concerns related to donor safety, graft integrity, and the need for rapid and reliable vascular and biliary anastomoses.

Minimally invasive approaches in donors have progressively gained interest because of the potential advantages including smaller skin incisions, shorter hospital stay, and reduced postoperative pain. After the first experiences with laparoscopic left lateral sectionectomy, mainly in the pediatric setting [73], hand-assisted and fully laparoscopic living donor right hepatectomy were subsequently introduced [74, 75]. The integration of robotic technology in LDH began in the early 2010s, further expanding the minimally invasive paradigm [2]. Subsequent studies suggested improved perioperative outcomes for minimally invasive LDH, including reduced blood loss and shorter length of stay compared with open surgery [3]. In a large single-center experience of 3448 cases, robotic LDH was associated not only with lower donor morbidity compared with laparoscopic and open approaches (4% vs. 8% vs. 16%, respectively, *p* < 0.001), but also with reduced recipient complications when grafts were procured robotically (23% vs. 44% vs. 31%, respectively, *p* < 0.001) [4]. Taken together, these findings suggest that minimally invasive LDH is no longer purely experimental, whereas robotic LDH may represent an advanced and promising evolution of this progressively consolidated minimally invasive pathway [5].

By contrast, the application of robotics to recipient LT remains in a much earlier and exploratory phase. The first fully robotic hemiliver transplantations following robotic LDH have been reported, demonstrating technical feasibility in highly selected cases [6]. More recently, fully robotic implantation of whole liver grafts from deceased donors has been achieved [76], with subsequent European experiences reported from pioneer centers [77, 78]. Furthermore, robotic implantation of grafts procured via robotic LDH has also continued to evolve, including reports of left hemiliver and dual-graft robotic LT [79].

Collectively, these experiences define the upper boundary of technical complexity currently achievable with robotic assistance. However, while robotic LDH is supported by an expanding comparative literature, fully robotic recipient LT should still be regarded as an early proof-of-concept application, limited to ultra-selected cases, highly specialized teams, and substantial logistical and technical resources. At present, these experiences underscore feasibility rather than a shift in standard clinical practice.

**Expert tips.** In robotic recipient LT, the reconstructive phase builds on the technical experience gained from isolated robotic vascular and biliary resections. Reconstruction should be started only after stable graft positioning and tension-free exposure have been achieved, while meticulous planning, experienced bedside assistance, and a predefined bailout strategy remain essential safety principles.

## Discussion

Robotic liver surgery has progressively evolved from a minimally invasive alternative for minor resections to a platform capable of supporting highly complex hepatobiliary procedures. The present review suggests that the added value of robotics lies primarily in its ability to facilitate technically demanding steps of liver surgery, especially when precise dissection and intracorporeal reconstruction are required in anatomically constrained settings. Beyond reconstruction, this also applies to oncologically relevant dissection steps, such as the clearance of periarterial lymphatic and neural tissue in advanced biliary malignancies requiring hilar or periarterial dissection.

Across the technical domains reviewed, the robotic platform appears particularly suited to procedures in which precision is critical. Enhanced dexterity, tremor filtration, stable three-dimensional visualization with magnification, and the possibility of seamlessly integrating adjunctive imaging may provide relevant technical advantages in selected cases. However, although current evidence consistently supports feasibility and short-term safety, clear superiority over laparoscopic surgery remains unproven, particularly with regard to long-term oncological outcomes.

A recurring theme across all domains of complexity is therefore the central role of training, case selection, and institutional experience. Procedures combining extreme technical demands with major physiological implications require not only advanced technology, but also structured training pathways, multidisciplinary expertise, and high institutional volume. In this context, robotic liver surgery should currently be regarded as a complementary tool within specialized hepatobiliary and transplant programs, rather than as a universally applicable solution.

Beyond technical feasibility, the diffusion of robotic liver surgery is also limited by important logistical and economic barriers. Although minimally invasive surgery is associated with significantly reduced hospital stay costs compared to open surgery, its procedural costs are significantly higher. In this context, laparoscopic liver surgery has lower procedural costs than robotic surgery, resulting in the overall least expensive approach among the three [80]. In addition, implementation requires access to robotic platforms, dedicated operating room logistics, specialized training for both surgeons and staff, and often an experienced bedside assistant. It is, however, reasonable to anticipate that procedural costs will progressively decrease over time, in parallel with the broader dissemination of robotic technology, increased market competition, and ongoing technological advancements.

Nevertheless, one of the distinctive strengths of the robotic platform may lie in its potential for technological integration. The ability to incorporate intraoperative ultrasound, fluorescence imaging, and, potentially, image-guided navigation, artificial intelligence, and augmented reality within a single operative interface may become increasingly relevant as liver surgery moves toward greater precision and personalization. Although many of these developments remain at an early stage, the current momentum in both technological innovation and clinical research is likely to further strengthen the role of robotics as a platform for image-integrated hepatobiliary surgery.

In conclusion, robotic liver surgery has reached a level of maturity that allows the safe execution of even the most complex hepatobiliary procedures in carefully selected settings. Its value lies in refining surgical performance and expanding the technical armamentarium of expert centers, rather than in redefining oncological or transplant indications. Future progress will depend not only on technological innovation, but also on standardization, training, and the generation of more robust comparative evidence.
